# A Novel Model Based on Genomic Instability-Associated Long Non-Coding RNAs for Predicting Prognosis and Response to Immunotherapy in Patients With Lung Adenocarcinoma

**DOI:** 10.3389/fgene.2021.720013

**Published:** 2021-10-29

**Authors:** Guangxu Tu, Weilin Peng, Qidong Cai, Zhenyu Zhao, Xiong Peng, Boxue He, Pengfei Zhang, Shuai Shi, Xiang Wang

**Affiliations:** ^1^ Department of Thoracic Surgery, The Second Xiangya Hospital, Central South University, Changsha, China; ^2^ Hunan Key Laboratory of Early Diagnosis and Precision Therapy of Lung Cancer, The Second Xiangya Hospital, Central South University, Changsha, China

**Keywords:** lung cancer, lung adenocarcinoma, long non-coding RNA, genomic instability, prognosis

## Abstract

**Background:** Emerging scientific evidence has shown that long non-coding RNAs (lncRNAs) exert critical roles in genomic instability (GI), which is considered a hallmark of cancer. To date, the prognostic value of GI-associated lncRNAs (GI-lncRNAs) remains largely unexplored in lung adenocarcinoma (LUAC). The aims of this study were to identify GI-lncRNAs associated with the survival of LUAC patients, and to develop a novel GI-lncRNA-based prognostic model (GI-lncRNA model) for LUAC.

**Methods:** Clinicopathological data of LUAC patients, and their expression profiles of lncRNAs and somatic mutations were obtained from The Cancer Genome Atlas database. Pearson correlation analysis was conducted to identify the co-expressed mRNAs of GI-lncRNAs. Gene Ontology and Kyoto Encyclopedia of Genes and Genomes enrichment analyses were conducted to determine the main biological function and molecular pathways of the differentially expressed GI-lncRNAs. Univariate and multivariate Cox proportional hazard regression analyses were performed to identify GI-lncRNAs significantly related to overall survival (OS) for construction of the GI-lncRNA model. Kaplan–Meier survival analysis and receiver operating characteristic curve analysis were performed to evaluate the predictive accuracy. The performance of the newly developed GI-lncRNA model was compared with the recently published lncRNA-based prognostic index models.

**Results:** A total of 19 GI-lncRNAs were found to be significantly associated with OS, of which 9 were identified by multivariate analysis to construct the GI-lncRNA model. Notably, the GI-lncRNA model showed a prognostic value independent of key clinical characteristics. Further performance evaluation indicated that the area under the curve (AUC) of the GI-lncRNA model was 0.771, which was greater than that of the TP53 mutation status and three existing lncRNA-based models in predicting the prognosis of patients with LUAC. In addition, the GI-lncRNA model was highly correlated with programed death ligand 1 (PD-L1) expression and tumor mutational burden in immunotherapy for LUAC.

**Conclusion:** The GI-lncRNA model was established and its performance was found to be superior to existing lncRNA-based models. As such, the GI-lncRNA model holds promise as a more accurate prognostic tool for the prediction of prognosis and response to immunotherapy in patients with LUAC.

## Introduction

Lung cancer is one of the most common malignancies and causes the largest number of cancer-related deaths globally (Bray et al., 2018; Siegel et al., 2019). Lung cancer is histologically heterogeneous with lung adenocarcinoma (LUAC) as the most common pathological subtype, accounting for approximately 40% of all lung cancer cases (Denisenko et al., 2018). Although substantial progress and advances have been made in both the diagnosis and treatment of LUAC (e.g., surgical resection, immunotherapy, chemotherapy, targeted therapy), which have greatly improved the clinical outcome of LUAC patients, the prognosis of LUAC is still far from satisfactory with a 5-years survival rate as low as 21% (Macheleidt et al., 2018). Therefore, the development of a reliable prognostic tool is needed to precisely identify high-risk patients, and the implementation of optimal interventions is of great significance to improve patient prognosis in LUAC.

Genomic instability (GI), usually referred to as the high frequency of genetic mutations, is a hallmark of cancer, and these mutations allow cancer cells to adapt to environmental stress and drive the development of more aggressive cancer cells (Lengauer et al., 1998; Negrini et al., 2010). Abnormal transcriptional or post-transcriptional regulation potentially leads to gene mutations and chromosomal aberrations such as cell cycle checkpoints, DNA replication, DNA repair, mitosis, and epigenetic regulation (Soca-Chafre et al., 2019; Tam et al., 2019). For example, the aberrant expression of cell cycle-associated genes, such as cyclins and cyclin-dependent kinases, causes chromosomal change and promotes tumor progression (Broustas and Lieberman, 2014). A previous study revealed that nearly 2.5% of cancers can be attributed to mutations in DNA repair genes (Parry et al., 2017). Notably, lung cancer has the second highest frequency of somatic mutations (Kandoth et al., 2013), with an abnormal number of chromosomes, namely aneuploidy, which is detected in more than 60% of patients with non-small cell lung cancer, and genomic duplication occurs in more than 40% of patients with lung cancer (Burgess et al., 2020). These previous findings indicate the pivotal role of GI in the development and progression of lung cancer.

Long non-coding RNAs (lncRNAs), an emerging class of ncRNAs (Mattick and Rinn, 2015), exert regulatory roles in both genomic stability and GI (Liu, 2016; Nair et al., 2020). For example, the lncRNA GUARDIN promotes the expression of telomeric repeat-binding factor 2 by competitively binding to microRNA-23a, thus maintaining genomic stability (Hu et al., 2018). LncRNA LINC00657 inhibits mitosis, DNA repair, and DNA replication via binding to PUMILIO protein, which is essential for the maintenance of genomic stability (Elguindy et al., 2019). In contrast, lncRNA CCAT2 promotes carcinogenesis and GI (Chen et al., 2020). Given that lncRNAs have unique roles in maintaining genomic stability and promoting GI, we hypothesized that GI-associated lncRNAs may have prognostic value in LUAC.

In this study, we comprehensively analyzed the gene expression profiles, somatic mutations, and corresponding clinical data of LUCA patients from The Cancer Genome Atlas (TCGA) database, with the aim of developing a novel GI-lncRNA prognostic model to better predict the clinical outcomes of LUAC patients.

## Materials and Methods

### Data Acquisition and Processing

The level 3 transcriptome profiles of 535 LUAC tissues and 59 histologically normal tissues, somatic mutation profiles of 561 LUAC samples, and corresponding clinicopathological data of 522LUAC cases were acquired from TCGA database. For transcriptome profiles, mRNA data and lncRNA data were separated into a mRNA expression matrix and lncRNA expression matrix. For somatic mutation profiles, total mutation frequency of each case and frequency of the mutant gene were computed in all LUAC cases. After the LUAC cases with a survival time less than 30 days or incomplete follow-up information were excluded from further analyses, 490 patients with LUAC were randomly allocated into two cohorts: a training cohort (*n* = 246) and testing cohort (*n* = 244). The training cohort was used to identify GI-lncRNAs independently associated with overall survival (OS) for development of the prognostic index model, while the testing and total cohorts were used for validation of the newly developed model. The clinicopathological characteristics of patients in the training and testing cohorts are presented in Table 1.

**TABLE 1:** Characteristics of patients in the training, testing, and total cohorts.

Covariates	Training cohort	Testing cohort	Total cohort	*p* Value
Age (%)				
≤65 years	116 (47.15%)	115 (47.13%)	231 (47.14%)	0.999
>65 years	124 (50.41%)	125 (51.23%)	249 (50.82%)	—
Unknown	6 (2.44%)	4 (1.64%)	10 (2.04%)	—
Gender (%)				
Female	127 (51.63%)	135 (55.33%)	262 (53.47%)	0.465
Male	119 (48.37%)	109 (44.67%)	228 (46.53%)	—
Clinical stage (%)				
Stage I-II	192 (78.05%)	186 (76.23%)	378 (77.14%)	0.704
Stage III-IV	50 (20.33%)	54 (22.13%)	104 (21.22%)	—
Unknown	4 (1.63%)	4 (1.64%)	8 (1.63%)	—
T stage (%)				
T1-2	212 (86.18%)	214 (87.7%)	426 (86.94%)	0.620
T3-4	33 (13.41%)	28 (11.48%)	61 (12.45%)	—
Unknown	1 (0.41%)	2 (0.82%)	3 (0.61%)	—
N stage (%)				
N0	162 (65.85%)	155 (63.52%)	317 (64.69%)	0.520
N1-3	77 (31.3%)	85 (34.84%)	162 (33.06%)	—
Unknown	7 (2.85%)	4 (1.64%)	11 (2.24%)	—
M stage (%)				
M0	160 (65.04%)	164 (67.21%)	324 (66.12%)	0.810
M1	13 (5.28%)	11 (4.51%)	24 (4.9%)	—
Unknown	73 (29.67%)	69 (28.28%)	142 (28.98%)	—

### Identification of GI-Associated lncRNAs in LUAC

To identify GI-lncRNAs in LUAC, we initially computed the total somatic mutations of each case and integrated them with the lncRNA expression matrix by the sample names. According to the mutator hypothesis-derived computational frame as previously described (Bao et al., 2019), we ranked the LUAC patients by total somatic mutations, and defined the top 25% of cases as the genome unstable-like (GU-like) group and the last 25% cases as the genome stable-like (GS-like) group. Then we performed differential expression analyses between the GU-like group and GS-like group using the “limma” R package, and lncRNAs with a false discovery rate (FDR) less than 0.05 and |logFC| > 1 were identified as GI-lncRNAs. A heatmap and volcano plot were constructed to visualize these differentially expressed lncRNAs using the “igraph” R package.

### Hierarchical Clustering Analysis of the Differentially Expressed lncRNAs

We performed hierarchical clustering analysis of GI-lncRNAs in the LUAC cases. Briefly, “sparcl,” “pheatmap,” and “limma” R packages were used to compute Euclidean distances, and LUAC cases were stratified into two clusters. Then the clusters matrix was integrated with total somatic mutations count matrix. By comparing the median mutation counts of two clusters, the cluster with lower mutation count was defined as the GS-like cluster, while the other was defined as the GU-like cluster. Then the difference in total somatic mutation counts between two clusters was explored using the “limma” R package. Given that ubiquilin-4 (UBQLN4) has been demonstrated to be a driver gene of GI and is overexpressed in malignant tumors (Jachimowicz et al., 2019), we compared the expression levels of UBQLN4 between the two clusters in this study.

### Gene Co-expression Network and Functional Enrichment Analysis

To reveal the potential biological function and molecular pathways of GI-lncRNAs, we performed gene co-expression analysis to identify their co-expressed mRNAs. Pearson correlation analysis was performed to identify mRNAs that were co-expressed with the GI-lncRNAs using the “limma” R package by the threshold of correlation coefficient >0.3 and *p* < 0.05. The top 10 co-expressed mRNAs were selected for subsequent analyses of the gene co-expression network using the “igraph” R package. Then Gene Ontology (GO) and Kyoto Encyclopedia of Genes and Genomes (KEGG) enrichment analyses were performed for the co-expressed mRNAs by the “clusterProfiler” R package. Molecular pathways with *p* < 0.05 were considered significantly enriched.

### Construction and Validation of the GI-Associated lncRNA Signature

The training cohort was used to identify GI-lncRNAs independently associated with OS for construction of the prognostic index model. In brief, univariate Cox regression analysis was performed to identify GI-lncRNAs significantly related to OS (*p* < 0.05) using the “survival” R package. These GI-lncRNAs were subsequently subjected to multivariate Cox proportional hazard regression analysis to construct the optimal prognostic model (termed GI-lncRNAsSig) using the “survival” and “survminer” R packages. Patients’ risk score of the GI-lncSig was calculated as follows: risk score = Σ (ExpmRNAn × βmRNAn). According to the median risk score, patients were stratified into high- and low-risk groups. Kaplan–Meier survival analysis and receiver operating characteristic (ROC) analysis were used to evaluate the performance of the GI-lncRNAsSig for the prediction of OS using the “survminer” and “survivalROC” R packages. Finally, the testing cohort was used to validate the prognostic performance of the developed GI-lncRNAsSig in patients with LUAC.

### Clinical Risk Stratification and Independent Prognostic Value of the GI-lncRNA-Based Model

To explore the applicability of the GI-lncRNA-based prognostic model, we performed clinical risk stratification in the total TCGA cohort. In brief, LUAC patients were stratified into subgroups based on clinical characteristics including age (≤65 and >65), gender (female and male), tumor stage (I–II and III–IV), pathologic T classification (T1-2 and T3–4), pathologic N classification (N0 and N1–3), and pathologic M classification (M0 and M1). Each subgroup was further stratified into high- and low-risk groups according to the median risk score of the newly developed GI-lncRNA-based model. Kaplan–Meier survival analysis was performed to explore the survival difference between the high- and low-risk groups. Multivariate Cox regression analysis was performed to determine whether the GI-lncRNA-based model could have independent prognostic value in the training, testing, and total cohorts.

### Statistical Analyses

Statistical analyses were conducted using R software version 4.0.2. The Mann–Whitney test was used to compare quantitative data between different groups, and the chi-squared test was used to compare categorical data between groups. *p* < 0.05 (if not specified) was considered statistically significant.

## Results

### Identification of GI-lncRNAs in LUAC

**FIGURE 1:**
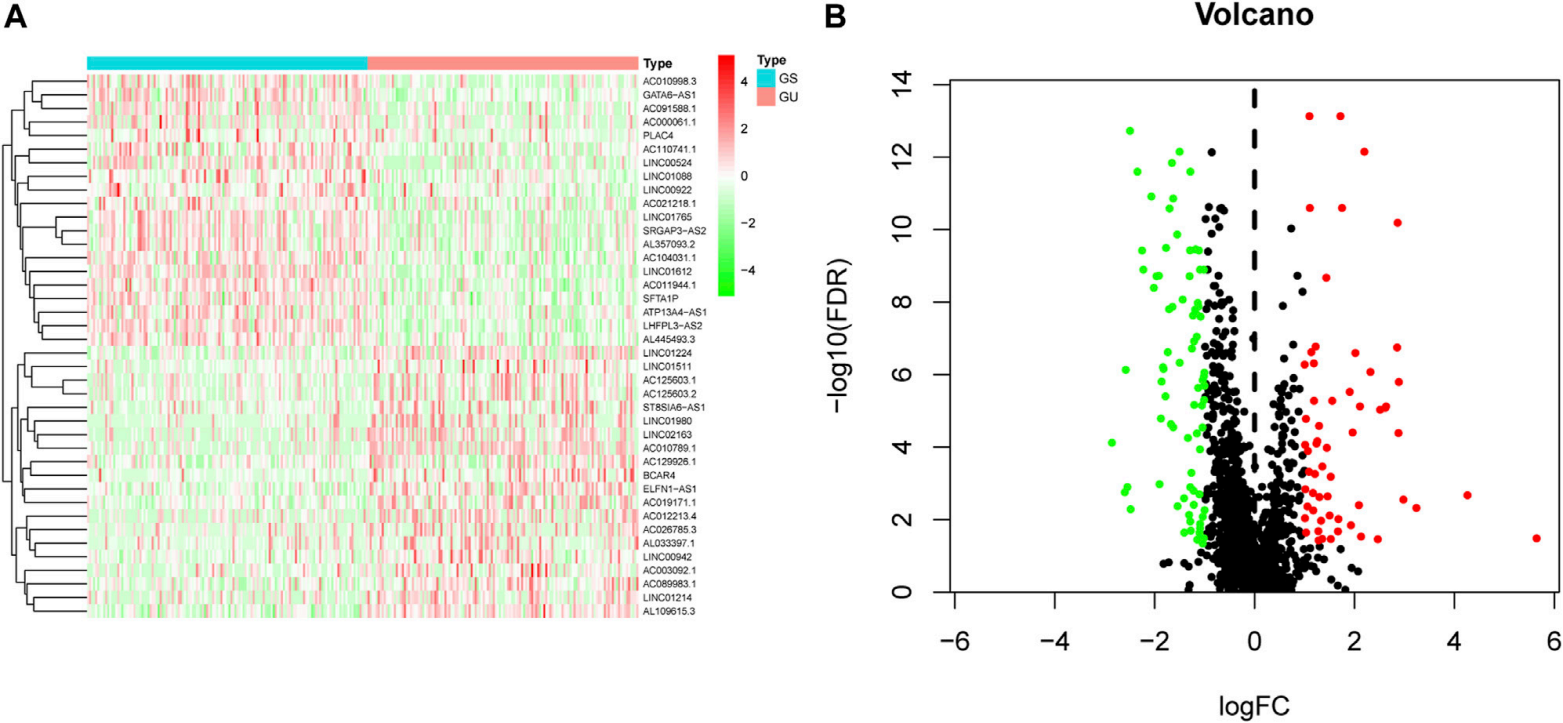
Heatmap and volcano plot of differentially expressed lncRNAs in LUAC. **(A)** Heatmap of differentially expressed lncRNAs in the GS-like group versus the GU-like group. The upregulated lncRNAs are denoted in red, and the downregulated lncRNAs are indicated in green; **(B)** Volcano plot of differentially expressed lncRNAs in the GS-like group versus the GU-like group. The upregulated lncRNAs are denoted in red, the downregulated lncRNAs are indicated in green, and the unaltered lncRNAs are denoted in black.

**FIGURE 2:**
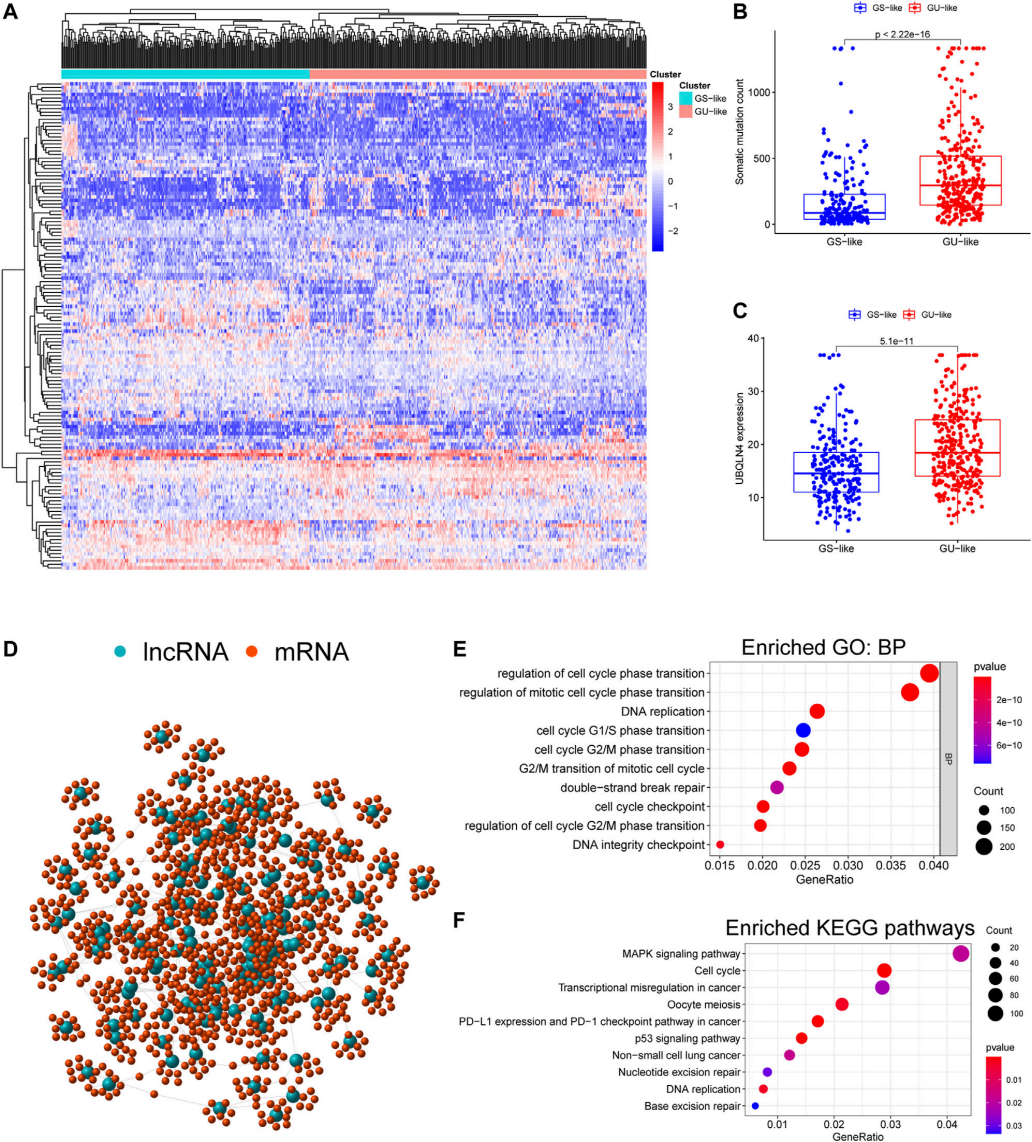
Functional annotation of GI-lncRNAs in LUAC patients. **(A)** Hierarchical clustering analysis of differentially expressed lncRNAs in the two clusters: GS-like cluster and GU-like cluster; **(B)** Somatic mutation counts in the GS-like cluster and GU-like cluster; **(C)** Comparison of expression levels of UBQLN4, a driver gene of GI, between the GS-like cluster and the GU-like cluster; **(D)** Network analysis of the relationship between GI-lncRNAs and mRNAs; **(E)** GO analysis; **(F)** KEGG pathway analysis.

We initially performed comprehensive analyses of the somatic mutation profiles of 561 LUAC cases, and the resulting data are presented in Supplementary Tables S1, S2 and Supplementary Figure S1. Somatic mutations were detected in the majority of LUAC tumor tissues (449/561, 88.95%) affecting 18,498 genes. The tumor protein p53 (TP53) (*n* = 272), giant-muscle filament titin (*n* = 265), mucoprotein-16 (*n* = 244), ryanodine receptor 2 (*n* = 228), and CUB and sushi multiple domains 3 (*n* = 215) were the top five most frequently mutated genes. After integrating the lncRNA expression matrix with the somatic mutations by the sample names, the top 25% cases with the highest mutation frequency were defined as the GU-like group, and the last 25% cases with the lowest mutation frequency were defined as the GS-like group. Differential analyses with the heatmap and volcano plot revealed that the expression levels of 138 lncRNAs were significantly different between the GU-like group and GS-like group, in which 59 were significantly upregulated and 79 were significantly downregulated (FDR <0.05, |logFC| > 1) (Figure 1). Based on the 138 differentially expressed lncRNAs, 490 LUAC patients were stratified into two clusters by hierarchical clustering analysis (Figure 2A). The cluster with the higher somatic mutation count was defined as the GU-like cluster, while the other was defined as the GS-like cluster. As shown in Figure 2B, the total somatic mutation count of the GU-like cluster was significantly higher than that of the GS-like cluster (*p* < 0.001). Notably, the expression of UBQLN4, a driver gene of GI, was significantly upregulated in the GU-like cluster (*p* < 0.001; Figure 2C). These results suggest that the 138 differentially expressed lncRNAs could be considered GI-lncRANs in LUAC.

### Functional Annotation and Molecular Pathway Analysis of GI-LncRNAs in LUAC

To reveal the potential biological functions and disrupted molecular pathways of the 138 GI-lncRNAs, we performed GO and KEGG enrichment analyses for their co-expressed mRNAs, which were identified by Pearson correlation analysis using a threshold of correlation coefficient >0.3 and *p* < 0.05. As shown in Figure 2D, a lncRNA-mRNA co-expression network was conducted using the GI-lncRNAs and their top 10 co-expressed mRNAs by the “igraph” R package. GO enrichment analysis indicated that the biological processes of the co-expressed mRNAs were mainly related to the formation of GI including cell cycle regulation, double-strand break repair, and DNA integrity checkpoint (Figure 2E). KEGG enrichment analysis indicated that these co-expressed mRNAs were enriched in molecular pathways associated with GI including cell cycle regulation, p53 signalling pathway, transcriptional dysregulation, nucleotide excision repair, and base excision repair (Figure 2F). Interestingly, programmed death-ligand 1 (PD-L1) expression and the programmed cell death protein 1 (PD-1) checkpoint pathway was enriched in these co-expressed mRNAs. Together, these results suggest that these 138 lncRNAs are closely associated with GI, and their aberrant expression may disrupt the lncRNA-mRNA regulatory network, thus promoting GI.

### Construction and Validation of the GI-lncRNA-Based Prognostic Model

**FIGURE 3:**
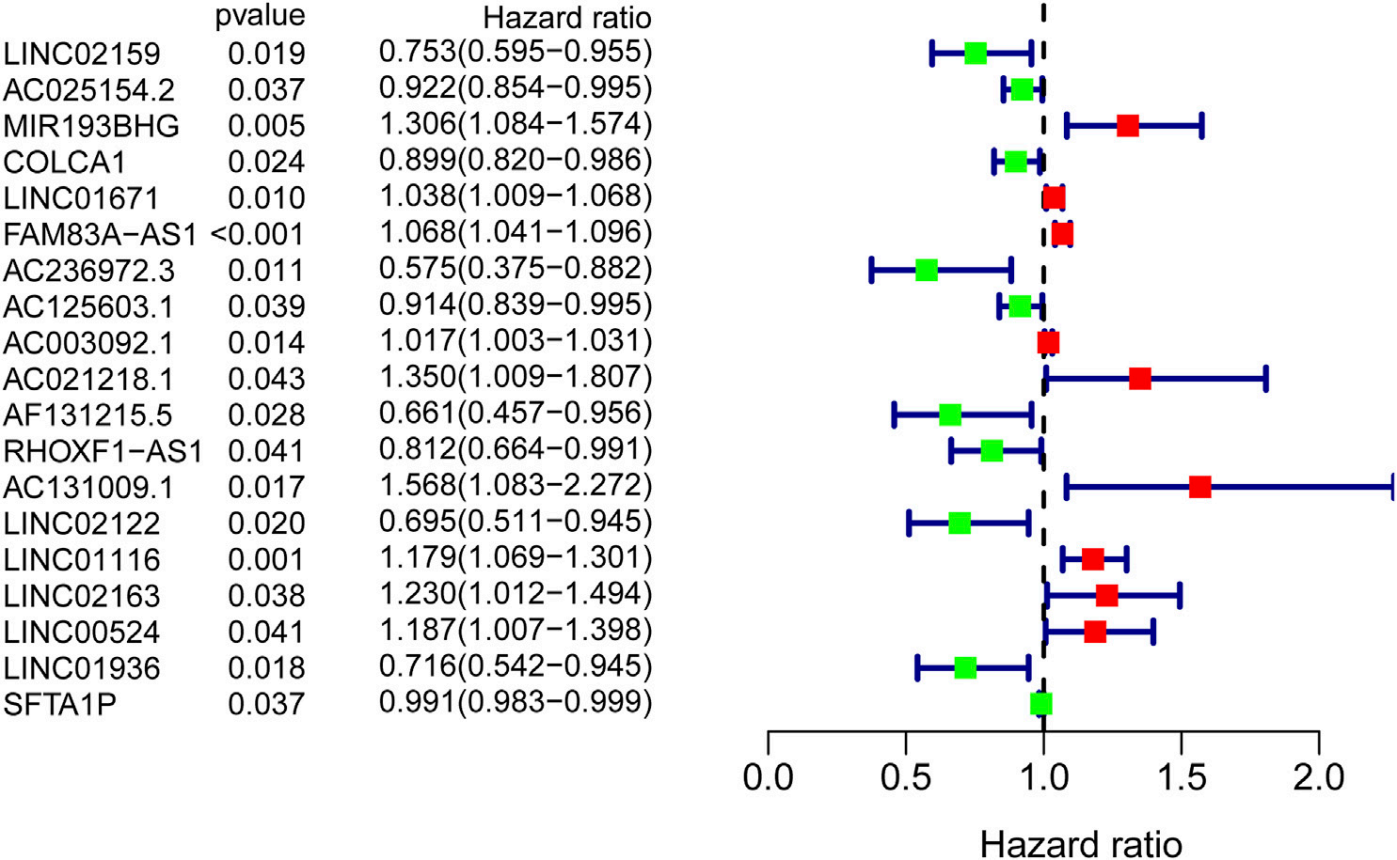
GI-lncRNAs associated with the prognosis of LUAC patients in the univariate Cox regression analysis. A total of 19 GI-lncRNAs were significantly associated with OS, including 9 risk factors for OS and 10 protective factors.

To identify GI-lncRNAs with prognostic value, univariate Cox regression analysis was performed in the training cohort. Among the 138 GI-lncRNAs, 19 were significantly associated with OS, including 9 risk factors for OS and 10 protective factors (Figure 3). Furthermore, multivariate Cox proportional hazard regression analysis identified nine GI-lncRNAs to construct the optimal prognostic model. The risk score to predict OS in LUAC patients was calculated as follows: risk score = (−0.192 * expression of LINC02159) + (−0.059 * expression of AC025154.2) + (0.049 * expression of LINC01671) + (0.051 * expression of FAM83A-AS1) + (−0.113 * expression of AC125603.1) + (0.423 * expression of AC021218.1) + (−0.409 * expression of AF131215.5) + −0.126 * expression of RHOXF1-AS1) + (0.151 * expression of LINC01116).

**FIGURE 4:**
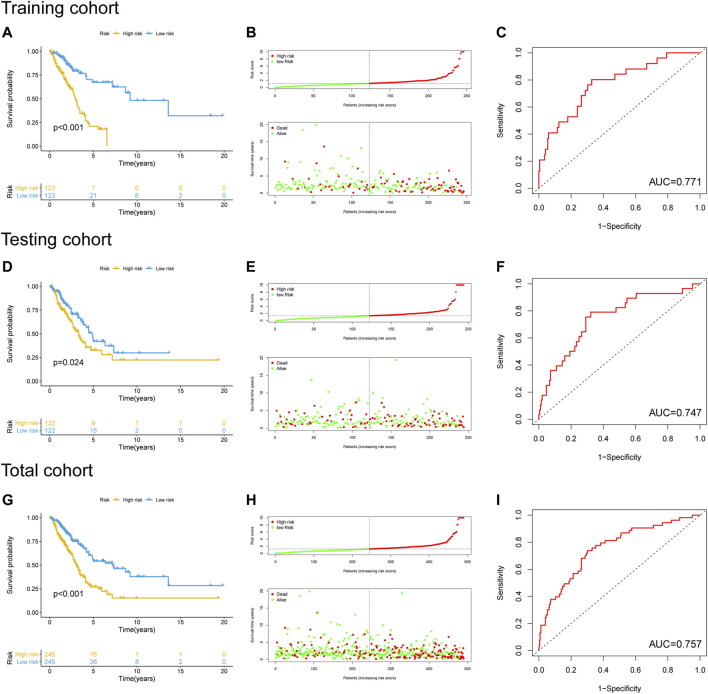
Performance evaluation and validation of the newly developed GI-lncRNA-based model for the prediction of OS in LUAC patients. Kaplan–Meier analysis of OS between the high-risk group and low-risk group in **(A)** the training cohort, **(D)** the testing cohort, and **(G)** the total cohort; The relationship between OS and risk-risk scores of the new prognostic model in the **(B)** the training cohort, **(E)** the testing cohort, and **(H)** the total cohort; The receiver operating curves for the new prognostic model in **(C)** the training cohort, **(F)** the testing cohort, and **(I)** the total cohort.

The performance of the newly constructed prognostic model was initially evaluated in the training cohort. After LUAC patients were stratified into high- and low-risk groups based on the median risk score of the prognostic model (Supplementary Table S3), Kaplan–Meier survival analysis showed a significant difference in OS between the groups (Figure 4A), with significantly poorer prognosis found in the high-risk group compared to the low-risk group (*p* < 0.001; Figure 4B). ROC curve analysis revealed that the area under the curve (AUC) value of the newly developed model was 0.771, indicating good performance in predicting the prognosis of patients with LUAC (Figure 4C). The prognostic model was validated using the testing cohort and total cohort in which LUAC patients were stratified into high- and low-risk groups in accordance with the median risk score of the prognostic model (Supplementary Tables S4, S5). Kaplan–Meier curve analysis indicated a significantly worse OS in the high-risk group (*p* = 0.024; Figure 4D) with the poorer OS correlated with higher risk scores (Figure 4E). The AUC value was 0.747. Similar results were obtained in the total cohort (Figures 4G–I). These results indicated that the performance of the newly develop GI-lncRNA-based prognostic model was well validated for the prediction of OS in patients with LUAC.

### Determination of the Independent Prognostic Value of the GI-lncRNA-Based Model for LUAC

**FIGURE 5:**
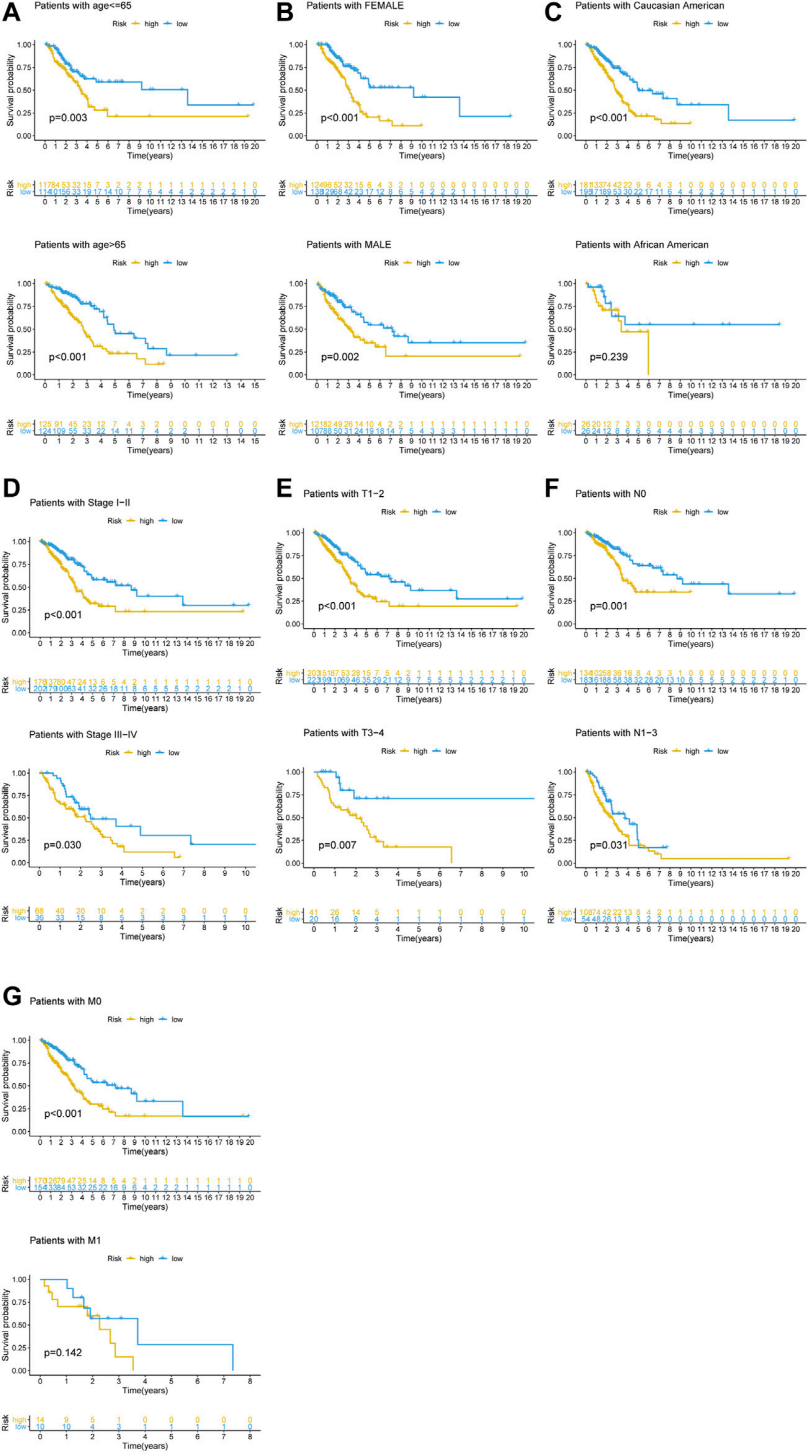
Kaplan–Meier analysis of OS between the high-risk and low-risk groups in LUAC patients with different clinical characteristics. Kaplan–Meier curves of LUAC patients with different clinical characteristics, including **(A)** age (≤65/>65); **(B)** gender (female/male); **(C)** race (Caucasian American/African American); **(D)** stage of lung cancer (Stage I-II/III-IV); **(E)** pathologic T stage (T1-2/T3-4); **(F)** pathologic N stage (N0/N1-3); **(G)** pathologic M stage (M0/M1).

To determine if the newly developed GI-lncRNA model could independently predict prognosis, we initially performed risk stratification analysis based on key clinical characteristics of LUAC patients and risk scores of the GI-lncRNA model. Patients were stratified into different subgroups by key clinical characteristics, including age (≤65 and >65), gender (female and male), tumor stage (I-II and III-IV), pathologic T classification (T1-2 and T3-4), pathologic N classification (N0 and N1-3), and pathologic M classification (M0 and M1). Patients in each subgroup were further stratified into the high- and low-risk groups according to the risk scores of the GI-lncRNA model. As shown in Figure 5, Kaplan–Meier analysis showed that the OS of patients in the high-risk group was significantly worse than that of the low-risk group in all subgroups (*p* < 0.05), except for patients in the M1 subgroup and patients in the racial group of African American, which could be due to the relatively small sample size of the two subgroups. However, the OS of the low-risk group was better than that of the high-risk group in both subgroups, whereas there was no significant difference. To further determine whether the GI-lncRNA-based prognostic model could independently predict OS, we performed univariate Cox regression and multivariate Cox regression analyses in the training cohort, testing cohort, and total cohort. As illustrated in Table 2, the GI-lncRNA-based prognostic model was independent of key clinical characteristics to predict OS in LUAC patients in the training, testing, and total cohorts (all *p* < 0.05).

**TABLE 2:** Univariate and multivariate analyses of risk factors associated with the prognosis of LUAC patients.

Variables		Univariate analysis				Multivariate analysis			
		HR	HR.95L	HR.95H	Pvalue	HR	HR.95L	HR.95H	Pvalue
Training cohort (*n* = 246)									
Age	—	0.998	0.974	1.022	0.842	—	—	—	—
Gender	Male/Female	0.941	0.577	1.535	0.808	—	—	—	—
Clinical stage	(III + IV)/(I + II)	1.629	1.293	2.051	<0.001	1.247	0.888	1.752	0.202
T stage	(T3+T4)/(T1+T2)	1.588	1.202	2.099	0.001	1.049	0.737	1.494	0.790
N stage	(N1+N2+N3)/N0	2.743	1.663	4.525	<0.001	2.207	1.193	4.082	0.012
M stage	M1/M0	1.977	0.840	4.649	0.118	—	—	—	—
Risk score	High/Low	1.283	1.194	1.379	<0.001	1.303	1.197	1.417	**<**0.001
Testing cohort (*n* = 244)									
Age	—	0.995	0.969	1.022	0.735	—	—	—	—
Gender	Male/Female	1.282	0.796	2.064	0.307	—	—	—	—
Clinical stage	(III + IV)/(I + II)	1.529	1.230	1.901	<0.001	1.223	0.885	1.692	0.223
T stage	(T3+T4)/(T1+T2)	1.657	1.228	2.235	0.001	1.330	0.967	1.828	0.079
N stage	(N1+N2+N3)/N0	2.579	1.588	4.187	<0.001	1.944	1.041	3.630	0.037
M stage	M1/M0	1.670	0.760	3.670	0.202	—	—	—	—
Risk score	High/Low	1.003	1.001	1.005	0.007	1.003	1.001	1.006	0.003
Total cohort (*n* = 490)									
Age	—	0.997	0.979	1.014	0.700	—	—	—	—
Gender	Male/Female	1.076	0.765	1.512	0.675	—	—	—	—
Clinical stage	(III + IV)/(I + II)	1.585	1.353	1.858	<0.001	1.268	0.879	1.830	0.205
T stage	(T3+T4)/(T1+T2)	1.588	1.301	1.939	<0.001	1.272	1.007	1.607	0.043
N stage	(N1+N2+N3)/N0	2.561	1.817	3.609	<0.001	1.813	1.078	3.047	0.025
M stage	M1/M0	1.897	1.068	3.371	0.029	0.982	0.403	2.393	0.969
Risk score	High/Low	1.003	1.001	1.005	0.004	1.003	1.001	1.006	0.002

### Performance Comparison of the Newly Developed GI-lncRNA Model With TP53 Mutation Status and Existing LncRNA-Based Models for Predicting the OS of LUAC Patients

**FIGURE 6:**
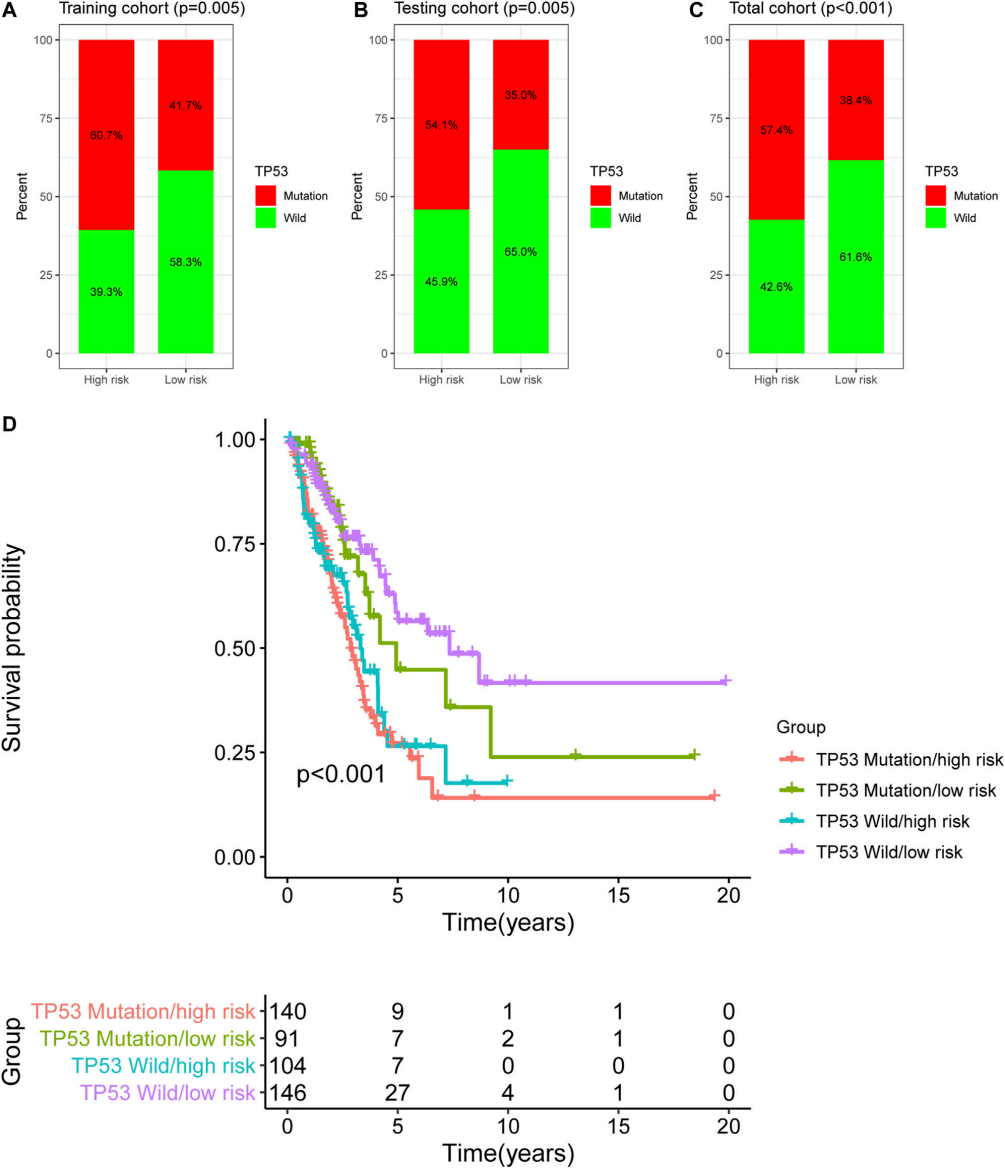
Comparison of the GI-lncRNA-based model and TP53 status for the prediction of OS in LUAC. Comparison of TP53 mutation status between the high-risk group and low-risk group stratified by the risk scores of the GI-lncRNA-based model in **(A)** the training cohort, **(B)** the testing cohort, and **(C)** the total cohort; **(D)** Kaplan–Meier analyses of LUAC patients in the following four groups stratified by the TP53 mutation status and risk score of the GI-lncRNA-based model: TP53 Wild/high risk group, TP53 Wild/low risk group, TP53 Mutation/high risk group, and TP53 Mutation/low risk group.

In this study, TP53 was found to be the most frequently mutated gene in LUAC (Supplementary Figure S1), therefore, we analyzed the TP53 mutation pattern between the high- and low-risk groups as stratified by the newly developed GI-lncRNA model. As shown in Figures 6A–C, the frequency of TP53 mutation was different in the high- and low-risk groups. In the training cohort, TP53 mutation was detected in 60.7% of patients in the high-risk group, which was significantly higher than the 41.7% in the low-risk group (*p* = 0.005; Figure 6A). Similar results were obtained in the testing cohort (Figure 6B) and total cohort (Figure 6C). Given that TP53 mutation status is closely related to GI and it has been proposed as a biomarker with prognostic value in lung cancer (Zhou et al., 2019; Freudenstein et al., 2020), we further evaluated the performance between the newly developed GI-lncRNAs and TP53 mutation status in predicting the OS of patients with LUAC. According to the TP53 mutation status and risk score of the GI-lncRNA model, LUAC patients were further classified into four groups: TP53 Wild/high risk group, TP53 Wild/low risk group, TP53, Mutation/high risk group, and TP53 Mutation/low risk group. As illustrated in Figure 6D, Kaplan–Meier analysis indicated the OS was significantly different among these four groups (*p* < 0.001). In the TP53 wild-type (WT) group, patients with a high risk score (referred to as TP53 Wild/high risk) had a worse OS than those with low risk score (referred to as Wild/low risk). In the TP53 mutation group, patients with a high risk score (referred to as TP53 Mutation/high risk) also had a worse OS than those with a low risk score (referred to as TP53 Mutation/low risk). In the low-risk group, patients with WT TP53 (TP53 Wild/high risk) had a better OS than those with TP53 mutation status (TP53 Mutation/high risk). However, in the high-risk group, the OS of patients with TP53 wild-type (TP53 Wild/high risk) was similar to those with TP53 mutation type (TP53 Mutation/high risk), indicating that TP53 mutation status failed to discriminate the OS of patients in the high-risk group. Interestingly, the OS of the TP53 Mutation/low risk group was better than that of the TP53 Wild/high risk group. Thus, these findings indicate the overall better prognostic value of the GI-lncRNA model than the TP53 mutation status.

**FIGURE 7:**
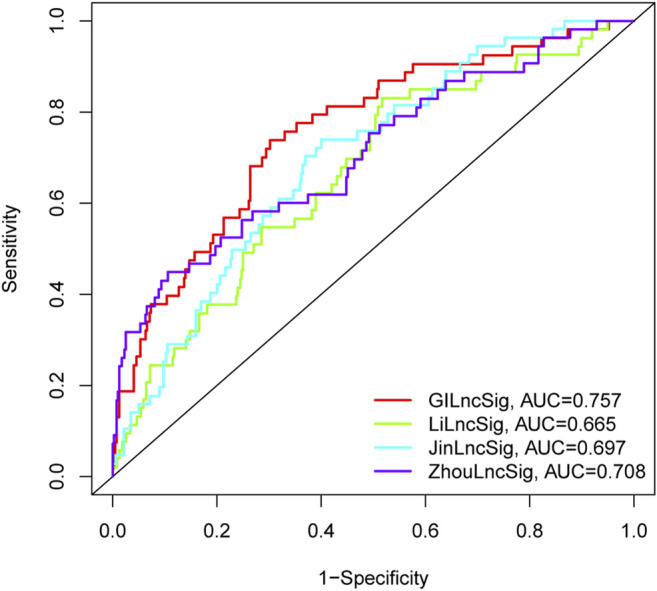
Performance comparison of the GI-lncRNA-based model with existing lncRNA-based prognostic models for LUAC. The receiver operating curves for the new prognostic model and the recently published lncRNA-based models for predicting the prognosis of LUAC patients.

We further conducted a performance comparison of the GI-lncRNAs with three recently published lncRNA-based prognostic models, including 7 lncRNA signatures from Li et al. (referred to as LiLncSig) (Li et al., 2020), 7 lncRNA signatures from Zhou *et al.* (referred to as JinLncSig) (Jin et al., 2020), and 13 lncRNA signatures from Zhou *et al.* (referred to ZhouLncSig) (Zhou et al., 2020). As shown in Figure 7, the AUC for the newly developed GI-lncRNA model was 0.757, which was significantly greater than that of LiLncSig (AUC, 0.653), JinLncSig (AUC, 0.696), and ZhouLncSig (AUC, 0.689). These data indicate that the newly developed GI-lncRNA model is superior to the three published lncRNA-based models.

### Value of the GI-lncRNA Model in Predicting Response to Immunotherapy

**FIGURE 8:**
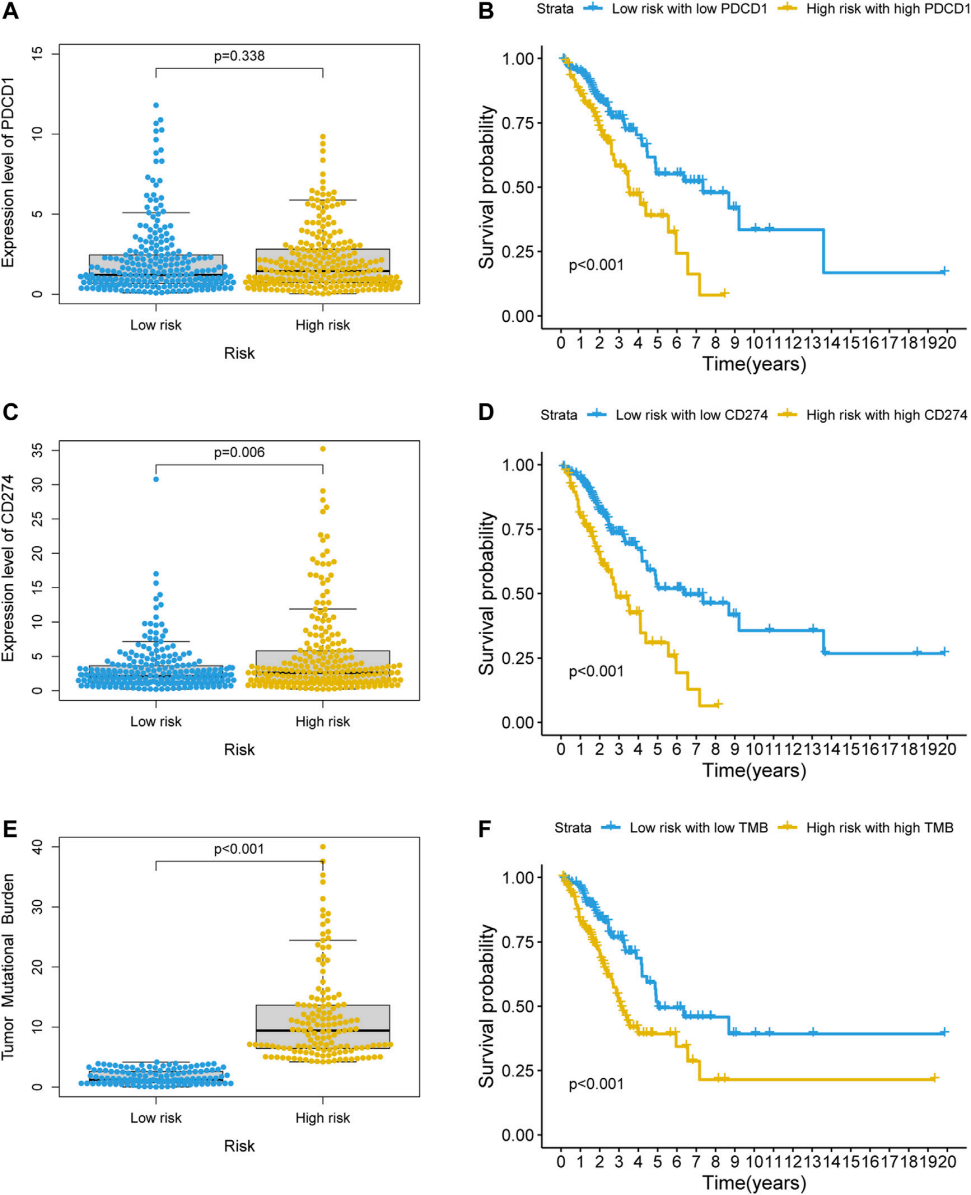
Value of the GI-lncRNA model for predicting the response to immunotherapy. Comparison of expression levels of **(A)** PDCD1, **(C)** CD274 between the low-risk and high-risk groups stratified by the risk scores of the GI-lncRNA-based model; Kaplan–Meier curve analysis of OS in **(B)** the low-risk group with low PDCD1 versus high-risk group with high PDCD1, and **(D)** low-risk group with low CD274 versus high-risk group with high CD274; **(E)** Comparison of difference in tumor mutational burden (TMB) between the high- and low-risk groups; **(F)** Kaplan–Meier analysis of OS in the low-risk group with low TMB versus high-risk group with high TMB.

KEGG enrichment analysis showed that PD-L1 expression and the PD-1 checkpoint pathway was enriched in the co-expressed mRNAs of GI-lncRNAs; thus, we further examined the expression pattern of PD-L1 and PD-1 in patients in different risk groups to explore the potential value of the newly developed GI-lncRNA model in predicting response to immunotherapy. As shown in Figures 8A,B, despite the significantly worse OS of patients in the high-risk and high PD-1 (PDCD1) group (*p* < 0.001), there was no significant difference in PD-1 expression between the high- and low-risk groups (*p* = 0.338), whereas the expression level of PD-L1 (CD274) in the high-risk group was significantly higher than that in the low-risk group (*p* = 0.006; Figure 8C) and the OS of the high-risk and high PD-L1 groups was significantly worse than that of the low-risk and PD-L1 groups (*p* < 0.001; Figure 8D). These findings indicate that the GI-lncRNA model can be used to predict the response to anti-PD-L1 immunotherapy. Combining tumor mutational burden (TMB) and PD-L1 expression greatly enhances the predictive power of response to immunotherapy efficacy (Hellmann et al., 2018). Given the hypothesis that patients with increased GI may have a higher frequency of somatic mutations and TMB, we further compared the difference in TMB between the high- and low-risk groups. Interestingly, the TMB of the high-risk group was significantly higher than that of the low-risk group (*p* < 0.001; Figure 8E), and Kaplan–Meier analysis indicated a significantly worse OS in patients with high TMB and in the high-risk group (*p* < 0.001; Figure 8F). In addition, we assessed the correlation between the GI-lncRNA model and PD-L1/TMB in the training cohort and testing cohort. As shown in Supplementary Figure S1, PD-L1/TMB was significantly higher in both cohorts. Therefore, LUAC patients in the high-risk group may benefit more from immunotherapy compared with those in the low-risk group, suggesting the value of the newly developed GI-lncRNA model for predicting the response to immunotherapy in patients with LUAC.

## Discussion

The prognostic value of GI-lncRNAs in patients with LUAC remains largely unexplored. The key novel findings of this study were as follows. Nineteen GI-lncRNAs associated with OS were identified in patients with LUAC. Nine GI-lncRNAs were significantly correlated with OS, which were used to generate the newly developed GI-lncRNA-based prognostic model. The GI-lncRNA model performed well with an AUC of 0.771, which was greater than the AUCs of the TP53 mutation status and three reported lncRNA-based models in predicting prognosis of patients with LUAC. The GI-lncRNA model showed a prognostic value independent of key clinical characteristics. The GI-lncRNA model was strongly correlated with PD-L1 and TMB, suggesting its value in predicting the response to immunotherapy in patients with LUAC. Together, these findings support the newly established GI-lncRNA model as a potentially better prognostic approach to predicting the prognosis and response to immunotherapy in LUAC patients.

Despite great advances in the diagnosis and treatment of lung cancer, OS is still considerably low and lung cancer remains the most common cause of cancer-related deaths globally (Lee et al., 2018). Traditional clinical parameters such as tumor-node-metastasis stage, tumor size, regional lymph node metastasis, and distant metastasis have long been used for prognosis prediction in lung cancer. However, these conventional prognostic factors are not always convincible, as patients with the same clinical and pathological characteristics may encounter completely different clinical outcomes (Liu et al., 2019). Therefore, a prognostic approach with high accuracy may assist clinicians in making optimal treatment decisions to improve the patient survival. The past several decades have witnessed great progress in understanding the biological mechanisms of tumorigenesis, development, and progression. One of the major breakthroughs was the involvement of GI in tumorigenesis and the therapeutic response (Negrini et al., 2010). Lung cancer has the second highest somatic mutation burden, just second to melanoma, indicating the potential prognostic and diagnostic value of GI (Kandoth et al., 2013). An increasing number of studies have suggested that the aberrant expression of lncRNAs plays a key role in GI (Liu, 2016; Nair et al., 2020). Therefore, identifying GI-lncRNAs in the whole genome and exploring their prognostic value are of great significance.

In this study, we identified 138 GI-lncRNAs in LUAC using the computational frame as previously reported by Bao et al. (2019). GO analysis showed that the biological processes of the co-expressed mRNAs of 138 lncRNAs were enriched in pathways of cell cycle regulation and DNA damage response, such as cell cycle checkpoint and double-strand break repair. Cell cycle checkpoints are the dynamically surveillance mechanism of major events in cell cycle that ensure the order, integrity and fidelity of cell replication, and dysregulation of cell cycle checkpoints that often accompany GI (Anand et al., 2020). For example, p53, a major cell cycle checkpoint of G2/M phase, plays an important role in DNA replication by halting the cell cycle at G2 phase and allowing the repair mechanism to restore genomic stability (Williams and Schumacher, 2016). Double-strand break repair is another important mechanism for maintaining genomic stability (Terasawa et al., 2014). In light of KEGG analysis, the co-expressed mRNAs were also enriched in molecular pathways of cell cycle regulation and DNA damage repair (e.g., cell cycle, transcriptional dysregulation in cancer, p53 signaling pathway, and nucleotide excision repair) in association with maintenance of genomic stability. These results further confirmed that these 138 lncRNAs were closely linked to GI. In addition, the co-expressed mRNAs were enriched in the MAPK signaling pathway, which is involved in cancer invasion and metastasis (Wei et al., 2017; Wen et al., 2019). Therefore, we postulated that the abnormal expression of these differentially expressed lncRNAs may disrupt the lncRNA-mRNA regulatory network, thereby leading to GI and promoting the invasive and metastatic capacity of tumor cells. Then we identified GI-lncRNAs related to OS and constructed a GI-lncRNA model consisting of nine lncRNAs to predict clinical outcomes in the training cohort. Among the nine GI-lncRNAs used for construction of the GI-lncRNA model, the biological functions of FAM83A-AS1 and LNC01116 have been well illustrated in LUAC. LncRNA FAM83A-AS1 promotes LUAC cell proliferation, migration, invasion and the epithelial–mesenchymal transition (EMT) by competitively combining miR-150-5p with MMP14, and Wang *et al.* reported that FAM83A-AS1 promotes the progression of LUAC by enhancing its pre-mRNA FAM83A via the ERK signaling pathway (Xiao et al., 2019). Zeng *et al.* reported that the overexpression of LINC01116 contributes to tumor proliferation and metastasis of LUAC cells, and Wang et al. reported that LINC01116 contributes to cisplatin resistance via the EMT process (Wang et al., 2020; Zeng et al., 2020). Although the biological functions of AF131215.5 and RHOXF1-AS1 have not been reported in cancer, our results are in accordance with previous findings showing that these two lncRNAs are associated with the OS of LUAC (Hou and Yao, 2021). The remaining five lncRNAs have not been reported in lung cancer or other human cancers. Their prognostic value and biological function need to be further investigated in future research. The GI-lncRNA model stratified LUAC patients into high- and low-risk groups with significantly different OS, and ROC curve analysis showed the high sensitivity and specificity of the GI-lncRNA model, which were further confirmed in the testing cohort and total cohort. Stratification analysis showed that the GI-lncRNA model was applicable for all clinical subgroups, and multivariate Cox regression analysis revealed that the GI-lncRNA model was an independent prognostic factor for OS in the training, testing, and total cohorts. These results indicate that the GI-lncRNA model may be a promising non-invasive biomarker for OS prediction in LUAC.

In the functional enrichment analysis, we found that the co-expressed mRNAs were enriched in the PD-L1 expression and PD-1 checkpoint pathway, and further analyses revealed higher PD-L1 expression with a significantly worse OS in the high-risk group, indicating that GI-lncRNAs may also have regulatory effects on PD-L1 expression. PD-L1 contributes to the immune evasion of tumor cells by binding to PD-1 and negatively modulating T-cell receptor signaling (Blank et al., 2005; Pardoll, 2012). In recent years, unprecedented achievements have been made in immune checkpoint inhibitors (ICIs) targeting PD-1 or its ligand PD-L1 such as nivolumab, atezolizumabm and pembrolizumab (Doroshow et al., 2021). In fact, PD-L1 expression is the only test approved by the U.S. Food and Drug Administration (FDA) for ICI first-line treatment decision-making in lung cancer (Borghaei et al., 2015). However, PD-L1 expression to predict immunotherapy has limitations (e.g., variability and intra-tumor heterogeneity) (McLaughlin et al., 2016). Moreover, patients with low or no expression of PD‐L1 may also have favorable responses to ICIs (Frigola et al., 2021). TMB is another promising biomarker for ICI response, which was approved by FDA for the treatment decision of pembrolizumab in 2020 (Subbiah et al., 2020). Though TMB and PD-L1 expression are unrelated, greater benefit was found in patients with high TMB and high PD-L1 expression treated with anti-PD-1 and anti-PD-L1 agents in lung cancer, indicating synergistic association of the two independent biomarkers (Carbone et al., 2017; Peters et al., 2017; Hellmann et al., 2018). Based on the hypothesis that accumulating genetic alterations resulting from GI may lead to a higher TMB, we further analyzed the pattern of TMB between different risk groups, and found a significantly higher TMB in the high-risk group. According to the above results, we infer that the GI-lncRNAs may have potential for selecting LUAC patients who will benefit more from immunotherapy.

This study had several limitations. First, the GI-lncRNA model was developed and validated with retrospective data from TCGA database. Although we tried to further validate it in the Gene Expression Omnibus (GEO) database, some of the lncRNAs in the GI-lncRNA model could not be found in the GEO datasets due to a limited number of lncRNAs conserved in the Affymetrix platform. Therefore, a prospective study in the real world will be required to verify its clinical application value. Second, the findings in this study could not explain how these lncRNAs affect GI and malignant behaviors of tumor cells, for which future in-depth mechanistic investigation is warranted to illustrate their biological function and underlying mechanism. Third, it is worth noting that the correlation between the GI-lncRNA model and TMB/PD-L1 expression was not experimentally verified in this study.

In conclusion, a novel prognostic model based on a panel of GI-lncRNAs was established in this study. Notably, the new prognostic model exhibits high prognostic accuracy and overall better performance compared with the reported lncRNA-based prognostic models. In addition, the GI-lncRNA model may help predict which LUAC patient may have a better response to immunotherapy. Therefore, this newly constructed prognostic model may assist oncologists/surgeons in navigating optimal treatment plans, thereby enhancing survival and eventually improving the care of LUAC patients.

## Data Availability

Publicly available datasets were analyzed in this study. This data can be found here: https://portal.gdc.cancer.gov/repository.
