# Supplementary figures and images for: A Novel Model Based on Genomic Instability-Associated Long Non-Coding RNAs for Predicting Prognosis and Response to Immunotherapy in Patients With Lung Adenocarcinoma
Source: Front Genet. 2021 Oct 29;12:720013. doi: 10.3389/fgene.2021.720013 (PMC8585772; doi:10.3389/fgene.2021.720013)

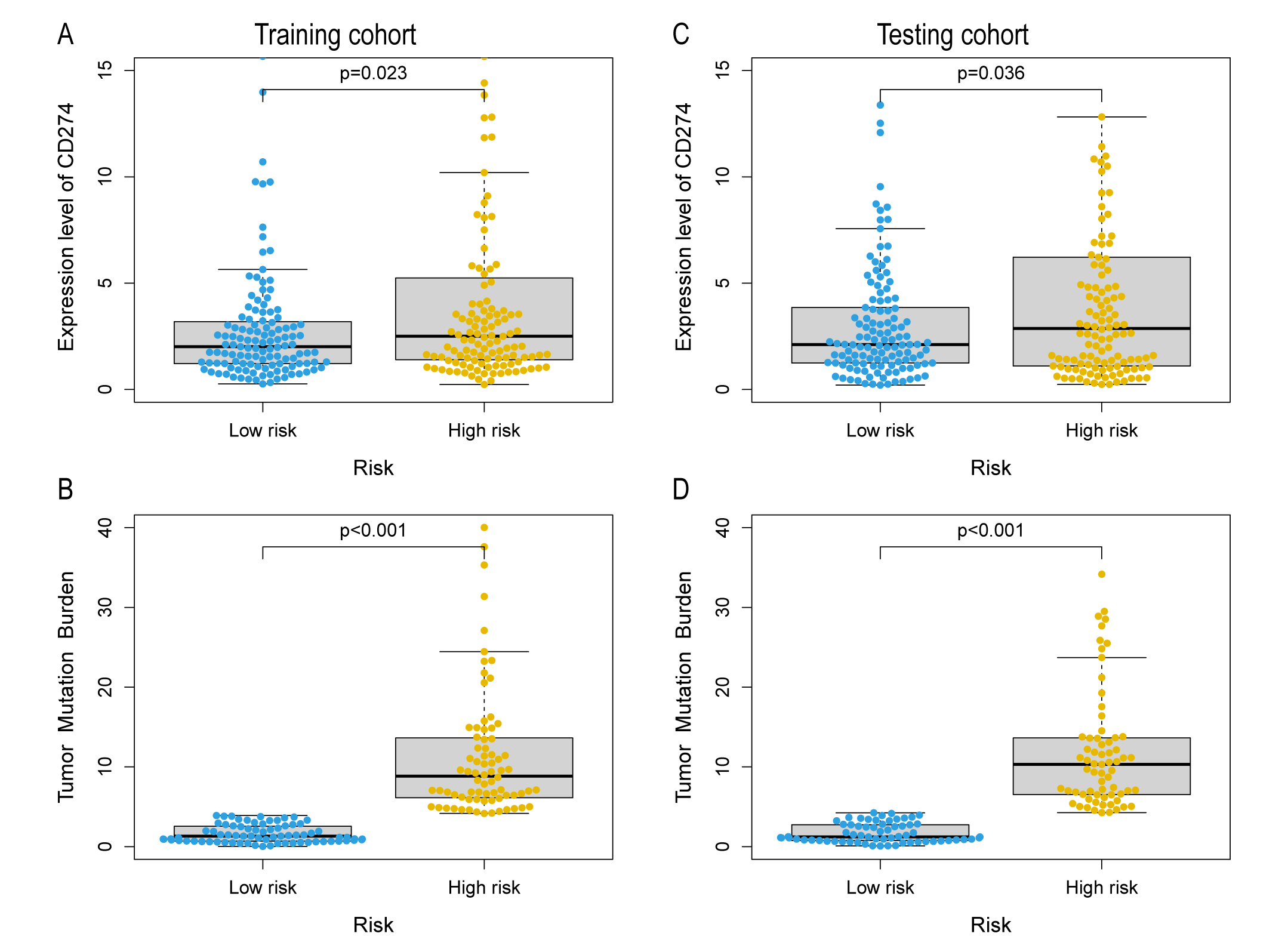

Supplement: Supplementary file 2 [file Image2.TIF]

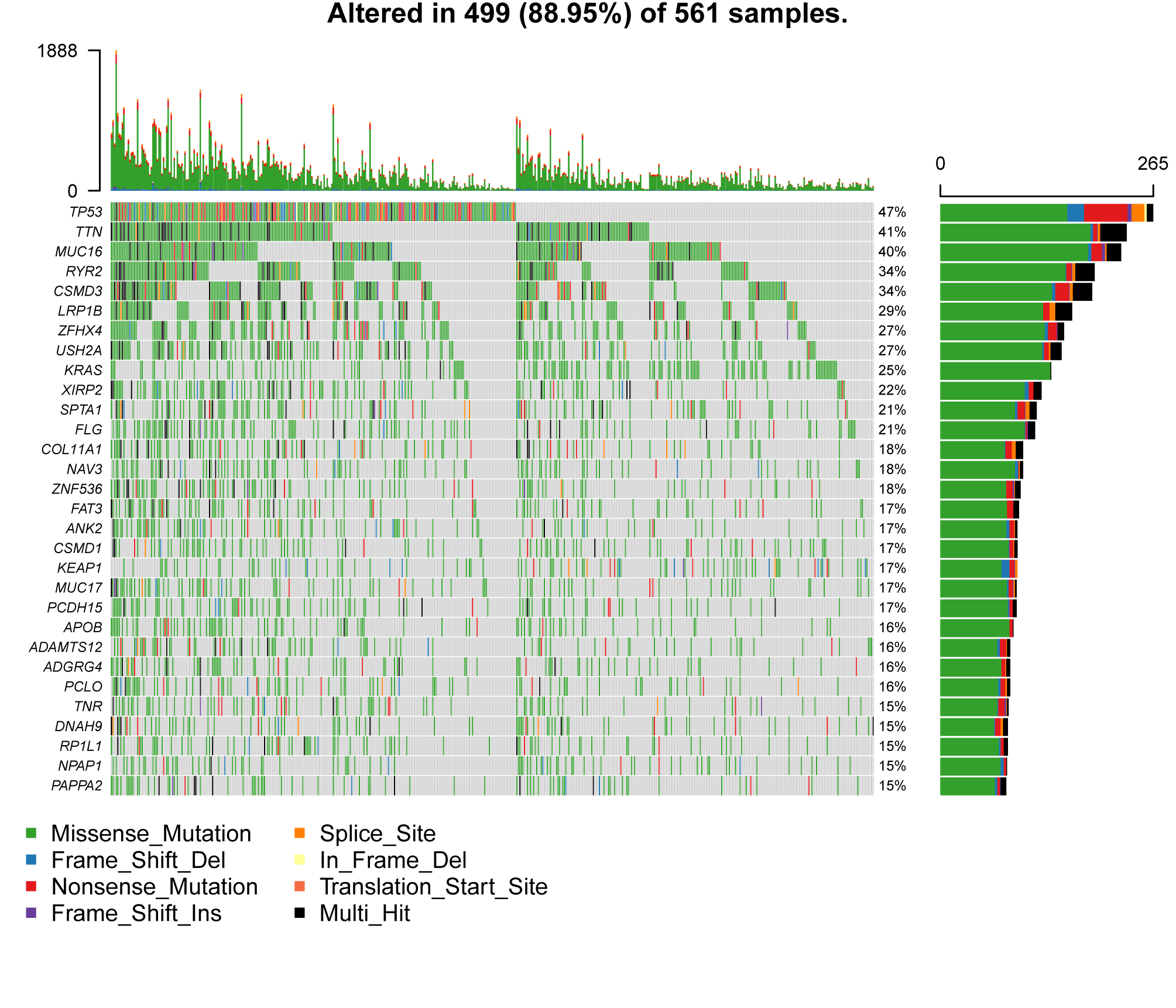

Supplement: Supplementary file 3 [file Image1.TIF]
